# Serum interleukin -8 is not a reliable marker for prediction of vesicoureteral reflux in children with febrile urinary tract infection

**DOI:** 10.1590/S1677-5538.IBJU.2014.0381

**Published:** 2015

**Authors:** Abolfazl Mahyar, Parviz Ayazi, Mohammad Hadi Yarigarravesh, Mohammad Hossein Khoeiniha, Sonia Oveisi, Ahmad Ali Sahmani, Shiva Esmaeily

**Affiliations:** 1 Department of Pediatrics, Qazvin Children hospital, Qazvin University of Medical Sciences, Qazvin, Iran; 2 Qazvin Children hospital, Qazvin University of Medical Sciences, Qazvin, Iran; 3 Diseases Research Center, Qazvin University of Medical Sciences, Qazvin, Iran; 4 Laboratory department of Qazvin Children hospital, Qazvin University of Medical Sciences, Qazvin, Iran; 5 Department of statistics, Qazvin University of Medical Sciences, Qazvin, Iran

**Keywords:** Vesico-Ureteral Reflux, Interleukin-8, Urinary Tract Infections

## Abstract

**Objective:**

In view of the side effects of voiding cystourethrography (VCUG), identification of noninvasive markers predicting the presence of vesicoureteral reflux (VUR) is important. This study was conducted to determine the predictive value of serum interleukin-8 (IL-8) in diagnosis of VUR in children with first febrile urinary tract infection (UTI).

**Materials and Methods:**

Eighty children with first febrile UTI were divided into two groups, with and without VUR, based on the results of VCUG. The sensitivity, specificity, positive and negative predictive value positive and negative likelihood ratio, and accuracy of IL-8 for prediction of VUR were investigated.

**Results:**

Of the 80 children with febrile UTI, 30 (37.5%) had VUR. There was no significant difference between the children with and without VUR and also between low and high-grade VUR groups in terms of serum concentration of IL-8 (P>0.05). Based on ROC curve, the sensitivity, specificity, likelihood ratio positive, and accuracy of serum IL-8 was lower than those of erythrocyte sedimentation rate and C-reactive protein. Multivariate logistic regression analysis showed significant positive correlation only between erythrocyte sedimentation rate and VUR.

**Conclusions:**

This study showed no significant difference between the children with and without VUR in terms of the serum concentration of IL-8. Therefore, it seems that serum IL-8 is not a reliable marker for prediction of VUR.

## INTRODUCTION

Urinary tract infection (UTI) is a common disease in children. Its prevalence in male and female children is 1% and 3–8%, respectively (1). Urinary tract infection occurs in three forms: acute pyelonephritis, cystitis, and asymptomatic bacteriuria. Acute pyelonephritis is the most severe type of the disease. The lack of early diagnosis and appropriate treatment result in dangerous complications, such as renal scarring (2, 3). The prevalence of renal scarring is 18–49% (2, 4–7).

The identification of risk factors in UTI is very important. The vesicoureteral reflux (VUR) is the most important risk factor of UTI. It corresponds to the retrograde flow of urine from the bladder to the ureter and, in some cases, to the pelvis and kidneys (2, 3). The prevalence of VUR in the first UTI is reported between 31.1–37.4% (8, 9). Unlike previous protocols, most current protocols advice towards a more selective use of voiding cystourethrography (VCUG) in children with first UTI, especially in children beyond infancy (10–12). Although VCUG can diagnose VUR, it is painful, invasive, and expensive and also iatrogenic UTI may ensue. Meanwhile, this method exposes children to radiation that may destroy gonads (13).

In view of the side effects of VCUG, lack of VUR in more than 50% of children with UTI, and spontaneous low-grade VUR recovery, researchers have been looking for cost-effective non-invasive predictors of VUR. Sun and et al. believed that serum procalcitonin is a suitable non-invasive marker for predicting VUR in children with UTI (14). We hypothesized that serum interleukin-8 is capable of predicting VUR in children with febrile UTI. Interleukin-8 is one of inflammatory cytokines that plays an important role in dealing with bacterial infections (15). Interleukin-8 was found to be elevated in the urine of children with infection or inflammation, so if a more intensified inflammatory response in children with VUR and UTI is regarded, then one could hypothesize a potential role of IL-8 not only locally (urothelium) but also at systematic level (15). The present study was conducted to determine the predictive value of serum IL-8 in diagnosis of VUR in children with the first febrile UTI.

## MATERIALS AND METHODS

This prospective cross-sectional study examined 80 children less than 12 years old diagnosed with first febrile UTI, in Qazvin's Children's Hospital affiliated to Qazvin University of Medical Sciences, Iran, in 2012–2013. The sample sized was calculated based on P=88% (sensitivity for urine IL-8), 1-P=12%, d=0.08, α=0.05 and 1-α=0.95 (15) and using the following equation:

The consecutive sampling was employed to achieve the required sample size.


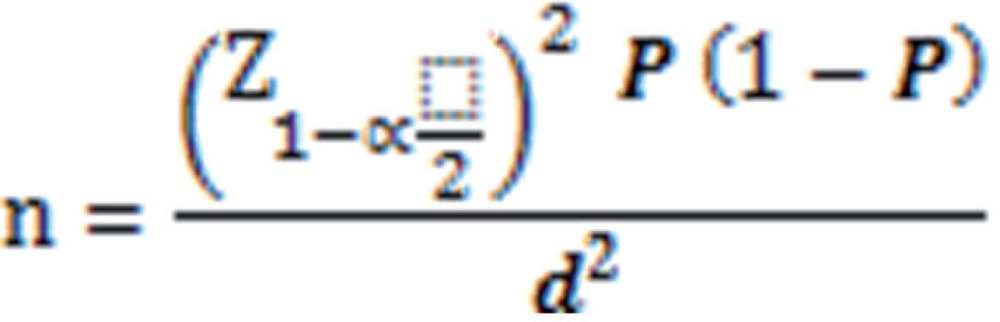


The inclusion criteria for children with febrile UTI were as follows: 1-presence of clinical symptoms, such as fever, poor feeding, anorexia, vomiting, and restlessness during urination in infants; and fever, vomiting, abdominal and flank pain, dysuria, and frequency in children; 2-abnormal urinalysis (leukocyturia, a positive nitrite test, etc.); 3-positive urine culture (the presence of more than 105 microorganisms of one kind per cc of urine using midstream or clean catch method, the presence of more than 104 microorganisms per cc of urine using catheterization method, or the presence of even one organism in the urine sample using suprapubic method) (2, 3); 4-the first UTI; and 5-undergoing VCUG. The children with: 1-lack of fever; 2-experiencing UTI more than once; 3-using antibiotics before admission, 4-accompanying and underlying diseases; 5-failure to undergo VCUG, and 6-suffering structural abnormalities of the urinary system (such as UPJO, neurogenic bladder, etc.) except VUR were excluded. Prior to the beginning of the antibiotic treatment, serum samples were send to the laboratory in order to test white blood cell count, neutrophil count, platelet count, erythrocyte sedimentation rate, and C-reactive protein quantitative level. The tests were performed in the laboratory of the children's hospital. To measure serum IL-8, 4 cc of blood was drawn from patients' peripheral vein. The serum was removed after centrifuging samples at 3000 rpm and 4ºC for 5 minutes. The serum was poured into acid-washed tubes and kept at-20°C until the test was performed. The serum IL-8 was measured using enzyme-linked immunosorbent assay (ELISA) method and AviBion Human IL-8 ELISA kit (Catalog no. IL08001, Orgenium Laboratories Business Unit, Orgenium Company, Finland). Based on the results of VCUG, the patients were divided into two groups: with VUR and without VUR. The diagnosis and grading of VUR were done according to International Study of Reflux in children (16). Grades 1 and 2 were regarded as low-grade VUR, and grades 3, 4, and 5 were regarded as high-grade VUR (17). Renal ultrasound was performed within the first 48 hours of admission. VCUG was performed at the end of the treatment when patients were discharged from the hospital. The 99mTc-dimercaptosuccinic renal scan was done in the first week of admission (3, 4). Ultrasound and VCUG were carried out by a radiologist, and renal DMSA scan was performed and interpreted by a nuclear medicine specialist. All patients were studied under similar conditions. Any report of hydronephrosis or hydroureteronephrosis without evidence of mechanical obstructions, such as UPJO, UVJO, and PUV, in the renal ultrasound, and any report of reduced uptake on the ^99m^Tc-dimercaptosuccinic renal scan for pyelonephritic changes in the kidneys were considered as suspicion for VUR (2, 3). The sensitivity, specificity, positive predictive value (PPV), negative predictive value (NPV), likelihood ratio positive (LRP), likelihood ratio negative (LRN), and accuracy of the clinical, laboratory, and imaging findings were determined for diagnosis of VUR. The data were analyzed using chi-square test, t-test, nonparametric tests (Mann-Whitney test), and multivariate logistic regression through SPSS15 software. P values less than 0.05 were considered significant.

## ETHICAL CONSIDERATION

The ethics committee of the Research Department in the Qazvin University of Medical Sciences (Project No.320) approved the study. All parents were provided information regarding the research method in simple language. The children were included in the study after their parents agreed and signed the informed consent form.

## RESULTS

Of the 80 children with the first febrile UTI, 10 (12.5%) and 70 (77.5%) children were male and female, respectively. Minimum and maximum age of the children were respectively 3 months and 132 months with median±IQR of 18.5±61.75 months. The most frequent symptoms were fever (100%), dysuria (77.5%) and frequency (77.1%). The most frequent microorganism grown in the urine culture was E-coli (91%). Of the 80 children with febrile UTI, 30 (37.5%) had VUR. Ratio of males/females in VUR and without VUR patients were 4/26 and 6/44, respectively (P=0.86). The minimum, maximum and median±IQR of age in the VUR and without VUR patients were 6, 132, 27±73 months and 3, 132, and 17.5±50.5 months, respectively (P=0.21).

The differences between the with and without VUR groups regarding clinical, laboratory and imaging findings are shown in Table-1. No significant difference was observed between low and high-grade VUR groups in terms of clinical, laboratory, and imaging variables (P>0.05). There was no significant difference between with and without VUR groups and also, low and high-grade VUR groups in terms of serum concentration of IL-8 (P>0.05) (Table-1). Based on cut-point values determined using ROC curve, sensitivity, specificity, LRP, and accuracy of serum IL-8 was lower than those of erythrocyte sedimentation rate and C-reactive protein.

**Table 1:** Comparison of clinical, laboratory and imaging findings between children with and without VUR.

Variables	VUR positive (n=30)	VUR negative (n=50)	P
Fever ≥ 38 0C	25(83.3) c	42(84)	0.93
Abdominal pain	15(50) c	8(16)	0.001
Flank pain	11(36.7) c	10(20)	0.1
Vomiting	16(53.3) c	29(58)	0.68
Frequency	26(86.7) c	31(62)	0.018
Urinary incontinence	17(56.7) c	10(20)	0.001
Dysuria	18(60) c	44(88)	0,004
Urgency	16(53.3) c	36(72)	0.09
Anorexia	11(36.7) c	26(52)	0.18
Irritability on micturation	1(3.3) c	0(0)	0.19
Neutrophil count (/mm^3^)	71.9±8.6 a	66.7±11.8	0.04
Platelet count (/mm^3^)	368433±82718 a	344955±91376	0.26
ESR (mm/h)	39.7±13.8 a	23.3±9.3	0.01
CRP (mg/dL)	66.8±16.2 a	48.3±21.5	0.01
Urine leukocyte/hpf	24.5(78.2)^b^	22(56.5)	0.71
Urine RBC/hpf	7(9)^b^	12(7.5)	0.058
Urine nitrite positive	22(73.3) c	41(82)	0.35
Urine leukocyte esterase positive	10(33.3) c	15(30)	0.75
Urine leukocyte cast	19(63.3) c	27(54)	0.41
Urine hyaline cast ≥ 1/LPF	10(33.3) c	13(26)	0.48
Urine RBC cast	3(10) c	3(6)	0.51
Ecoli/Other bacteria	25(83.3) c	48(96)	0.052
US findings suggestive of VUR	26(86.7) c	25(50)	0.001
DMSA findings suggestive of VUR	27(90) c	20(40)	0.01
IL8 (pg/mL)	12.1(122) c	33.5(84.6)	0.38

a Mean±SD (T-test);

b Median±IQR (Mann-Whitney test);

c Chi-square test

**Figure 1:**
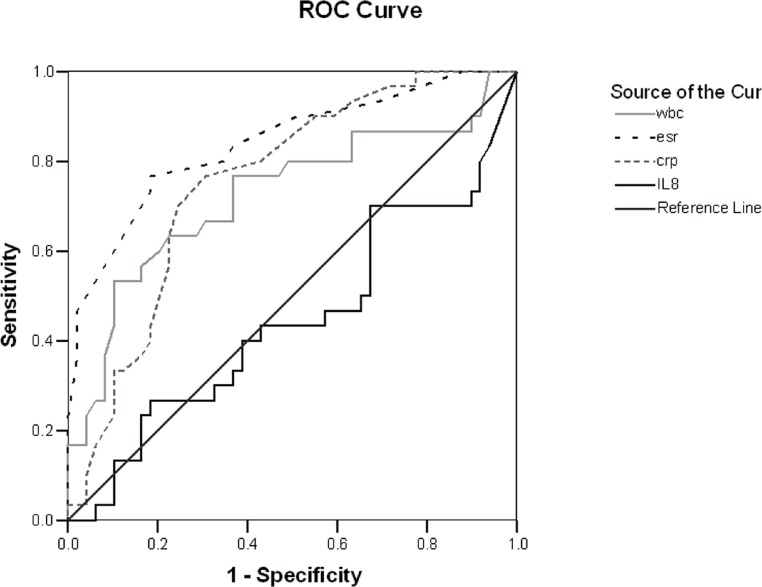
ROC curve for specificity and sensitivity of WBC and CRP and ESR and IL8 measurements. Area under ROC curve for WBC was 0.725 (95% CI 0.601–0.849 P=0.001, for ESR 0.840, 95%CI 0.747–0.934 P=0.0001, for CRP 0.755 95%CI 0.648–0.862 P=0.0001 and for IL-8 0.443 95%Cl 0.307–0.579 P=0.396).

Maximum sensitivity, likelihood ratio positive, and accuracy were related to ESR≥31mm/h, and maximum specificity was related to CRP≥43mg/dL (Table-2). Sensitivity, specificity, likelihood ratio positive, and accuracy of serum IL-8≥3.8pg/mL were 70, 32, 1.02, and 46, respectively (Table-2, Figure-1). Multivariate logistic regression analysis of VUR as the dependent variable and WBC count, platelet count, neutrophil counts, ESR, CRP, IL-8, ^99m^Tc-dimercaptosuccinic renal scan and other variables as independent variables revealed a significant positive correlation only between ESR and VUR (95% CI: 1.06–1.54, Beta=0.25, Odds Ratio=1.28, P=0.008).

**Table 2:** Sensitivity, Specificity, P.P.V and N.P.V of variables according ROC Curve.

Variables	Sensitivity (%)(95%CL)	Specificity (%)(95%CL)	PPV (%)(95%CL)	NPV (%)(95%CL)	LRP	LRN	Accuracy %
Serum interleukin 8≥8pg/mL	70(53–86)	32(19–44)	38(25–51)	64(45–82)	1.02	0.93	46
WBC count≥14800/mm^3^	76(61–91)	64(49–76)	56(40–71)	81(69–93)	2.08	0.36	68
Neutrophil count≥64.5%/mm^3^	80(65–94)	39(25–52)	44(31–57)	76(59–92)	1.3	0.51	54
Platelet count≥359000/mm^3^	60(42–77)	49(34–63)	44(29–59)	64(48–80)	1.17	0.81	53
ESR≥31mm/h	76(61–91)	82(71–92)	72(56–87)	85(75–95)	4.2	0.28	80
CRP≥43mgdL	90(79–100)	43(28–55)	48(35–61)	87(74–100)	1.55	0.23	60
Urine leukocyte/≥23hpf	57(38–76)	59(43–73)	47(29–64)	68(53–83)	1.39	0.72	58
Urine RBC≥6/hpf	56(32–80)	24(3–43)	41(20–61)	36(7–64)	0.73	1.85	39

## DISCUSSION

This study showed that serum IL-8 is not a reliable marker for prediction of VUR. The importance of diagnosing VUR is definitely clear for preventing renal damage in children (2, 3). Different methods are used to identify VUR, including VCUG, direct radionuclide cystography, radionuclide cystography, voiding uro-sonography and magnetic resonance voiding cystography (18, 19). VCUG is considered the gold standard investigation for VUR. Unfortunately, this technique has drawbacks. It requires urethral catheterization, which causes pain, risk of infection, and the use of radiation (20).

Due to the complications of VCUG, researchers have been looking for a noninvasive serum marker for predicting VUR (13). The noninvasive serum markers that have been studied are procalcitonin and acute phase reactants (14, 17). According to literature review and as far as our knowledge is concerned the present study is the first one that has surveyed the value of serum IL-8 in prediction of VUR in children with febrile urinary tract infection.

Sun and et al. reported that serum procalcitonin is useful for diagnosing acute pyelonephritis and predicting dilating VUR in young children with a first febrile urinary tract infection. They mentioned that a voiding cystourethrography is indicated only in children with high procalcitonin values (≥1.0 ng/mL) and/or abnormalities found on a ultrasonography (14). These results were confirmed in another study (21). Leroy and et al. reported that serum procalcitonin is a sensitive and validated predictor strongly associated with VUR≥3, regardless of the presence of early renal parenchymal involvement in children with the first UTI (21).

Soylu and et al. reported that serum CRP>50mg/l seems to be a potentially useful predictor of VUR and high-grade VUR (17). A few studies have been conducted about the role of urinary IL-8 in prediction of VUR, but the results are contradictory (22–23). In our study, sensitivity, specificity, LRP, and accuracy of serum IL-8 were lower than those of other markers especially erythrocyte sedimentation rate and C-reactive protein. In addition, there was a significant positive correlation only between ESR and VUR.

These findings mean that serum IL-8 is not a good predictor of VUR in children with children with first UTI. The reason may be that IL-8 probably acts mainly locally and not systematically during acute UTI even in more intensified inflammatory status, thus its serum levels are not significantly different. According to previous studies, IL-8 seems to be important at younger ages and the wide age-range of the present study might have affected the results (22, 23). Also, inclusion of a much smaller number of boys may be a potential confounding factor of the results. Interleukin-8 is a chemokine produced by macrophages and other cell types such as epithelial cells and endothelial cells in response to inflammatory processes (24). A trace amount of this cytokine exists in healthy people's urine (22, 23). Our limitation was lack of measurement of other cytokines such as IL-6.

## CONCLUSIONS

This study showed no significant difference between the children with and without VUR in terms of serum concentration of IL-8. Therefore, it seems that serum IL-8 is not a reliable marker for prediction of VUR.

## ABBREVIATIONS

UTI: = Urinary tract infection
VUR: = Vesicoureteral reflux
VCUG: = Voiding cystourethrography
